# Tuning Ultra‐Narrow Direct Bandgap in α‐Sn Nanocrystals: A CMOS‐Compatible Approach for THz Applications

**DOI:** 10.1002/smll.202509166

**Published:** 2025-11-02

**Authors:** Tiziano Bertoli, Elena Stellino, Francesco Minati, Camilla Belloni, Giovanni Tomassucci, Emanuele Bosco, Silvano Battisti, Leonardo Puppulin, Davide Cristofori, Vittorio Morandi, Francesca Rossi, Demetrio Logoteta, Alessandro Nucara, Luisa Barba, Gaetano Campi, Naurang Lal Saini, Fabrizio Palma, Pietro Riello, Michele Back, Fernanda Irrera

**Affiliations:** ^1^ DIET Sapienza University of Rome Via Eudossiana 18 Rome 00184 Italy; ^2^ Research Center for Nanotechnology for Engineering of Sapienza (CNIS) Sapienza University of Rome Piazzale Aldo Moro 5 Rome 00185 Italy; ^3^ Department of SBAI Sapienza University of Rome Via Antonio Scarpa 14 Rome 00185 Italy; ^4^ Department of Physics Sapienza University of Rome P.le Aldo Moro 2 Rome 00185 Italy; ^5^ Department of Molecular Sciences and Nanosystems Università Ca’ Foscari Venezia Via Torino 155 Mestre‐Venezia 30170 Italy; ^6^ CNR Institute for Nanostructured Materials (ISMN) Bologna Section, Via Piero Gobetti 101 Bologna I‐40129 Italy; ^7^ IMEM‐CNR Institute Parco Area delle Scienze 37/A Parma 43124 Italy; ^8^ Institute of Crystallography CNR Sincrotrone Elettra Strada Statale 14, Km 163.5, Area Science Park, Basovizza Trieste 34149 Italy; ^9^ Institute of Crystallography CNR Via Salaria Km 29.300, Monterotondo Roma 00015 Italy

**Keywords:** CMOS compatible process, nanocrystals, quantum confinement, THz applications, ultra‐narrow bandgap, α‐Sn

## Abstract

α‐Sn has recently been attracting significant interest due to its unique electronic properties. However, alternative strategies to the conventional epitaxial growth on InSb to stabilize it at room temperature and the ability to manipulate its bandgap are still a challenge. In this work, a complementary metal oxide semiconductor (CMOS)‐compatible process employing microwave irradiation is used to synthetize α‐Sn nanoparticles (NPs) of different size on a Si substrate. Morphological characterizations suggest the possibility to control the average Sn NPs size by means of a combined dewetting and coalescence process induced by the microwaves on Sn films. Transmission Electron Microscopy (TEM) and Synchrotron Radiation‐Grazing Incidence X‐ray Diffraction (SR‐GIXRD) analyses confirm the stabilization of the α‐Sn phase within an oxide shell, while X‐ray Photoelectron Spectroscopy (XPS) measurements allow tracking the oxide shell evolution and reveal the opening of a bandgap. Optical investigation demonstrates unprecedented tunability of the ultranarrow bandgap energy of α‐Sn between 64 and 137 meV (15–35 THz). The observed bandgap modulation with NPs size is consistent with a quantum confinement effect, which suggests the proposed approach as an effective strategy for tuning the α‐Sn bandgap and broadening its potential for a CMOS‐compatible integration in next‐generation terahertz technologies.

## Graphical abstract

α‐Sn NPs of different sizes are stabilized on Si wafer by means of a CMOS‐compatible process employing microwave irradiation. Unprecedented tunability of the ultranarrow bandgap energy of α‐Sn between 64 and 137 meV (15–35 THz) due to quantum confinement is demonstrated.

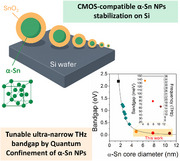

## 1 Introduction

Group IV semiconductors have been attracting considerable attention due to their intrinsic potential for integrated technologies compatible with the CMOS paradigm based on Si.^[^ ^1^, ^2^, ^3^ ^]^ After the pioneering work of He and Atwater in 1997,^[^ ^4^ ^]^ in the last few years the possibility of inducing a direct bandgap in Ge_1‐x_Sn_x_ thin films, nanowires, and nanocrystals^[^ ^1^, ^5^, ^6^, ^7^, ^8^, ^9^, ^10^, ^11^, ^12^ ^]^ has been widely investigated. Despite the large lattice differences between Si, Ge, and Sn, recent advances in synthesis techniques have enabled the stabilization of compounds with narrow direct bandgaps (*E_g_*) down to ≈0.3 eV^[^ ^13^ ^]^ (corresponding to a wavelength of ≈4 µm) by closing the bandgap of Si and Ge alloyed with Sn. Less attention has been paid to the alternative approach of opening an ultra‐narrow bandgap (*E_g_* < 250 meV) in α‐Sn, which could be exploited to bridge the THz gap,^[^ ^14^ ^]^ with potential applications in a variety of fields, ranging from the next generation mobile telecommunication system (5G, 6G)^[^ ^15^ ^]^ to quantum cybersecurity^[^ ^16^ ^]^ and astrophysics.^[^ ^17^ ^]^

At ambient pressure, tin exists in two main polymorphic structures: the thermodynamically stable tetragonal β‐Sn (space group *I41/amd*) and the diamond‐like cubic α‐Sn (space group *Fd‐3m*) stable below 13 °C.^[^ ^18^, ^19^, ^20^, ^21^, ^22^, ^23^, ^24^ ^]^ β‐Sn is a metal, while the electronic structure of α‐Sn is characterized by an inverted band order consisting of an upper inverted light hole (iLH) $\Gamma_{8}^{+}$ conduction band and a lower heavy‐hole (HH) $\Gamma_{8}^{+}$ valence band.^[^ ^25^, ^26^ ^]^ As a result, α‐Sn is semimetallic^[^ ^22^, ^27^ ^]^ and under strain it hosts topologically protected Dirac states.^[^ ^28^, ^29^, ^30^, ^31^ ^]^ This unique electronic structure potentially allows to tune the bandgap in the range of a few hundred meV, an energy window usually unavailable. However, the stabilization and control of the α‐Sn properties at operating temperatures remains a major obstacle for practical applications. Numerous studies have demonstrated stabilization at room temperature of α‐Sn films grown by molecular beam epitaxy (MBE) on substrates with diamond cubic cell structures, as GaAs, InSb, CdTe. It is speculated that the stabilization of the 𝛼‐phase occurs thanks to the layer‐by‐layer epitaxial growth on these types of substrates.^[^ ^29^, ^30^, ^32^, ^33^ ^]^ Other authors reported the stabilization of α‐Sn on silicon substrates covered by a bilayer composed by thin Ge film and a thin Sn film on top, after rapid thermal annealing and quenching. The observed diffusion of ≈2–4% Ge atoms in α‐Sn was hypothesized to trigger the α‐Sn stabilization by introducing mechanical stress.^[^ ^34^, ^35^ ^]^ However, among the strategies proposed for the stabilization of the α‐Sn, the effect of size is still debated. Some authors report the stabilization of the cubic α‐Sn phase in small structures,^[^ ^36^, ^37^ ^]^ in agreement with the general rule of the stabilization of the high symmetry phases already demonstrated in other materials such as CdSe, ZrO_2_, Ga_2_O_3_, BaTiO_3_, Al_2_O_3_.^[^ ^38^, ^39^, ^40^, ^41^ ^]^ On the contrary, other studies provided evidence of the stabilization of the tetragonal β‐Sn allotrope when the size decreases.^[^ ^42^ ^]^ Furthermore, despite the extensive investigation of the physical properties of α‐Sn grown on InSb, the control of its bandgap in an ultra‐narrow energy range is still a significant challenge.

In this study, we demonstrate the possibility to control the size of Sn NPs on Si substrate by using Sn films of different thickness and to stabilize the cubic α‐Sn structure by means of a patented microwave (MW) irradiation‐based CMOS‐compatible process.^[^ ^43^, ^44^ ^]^ The NPs formation process and the effect of their size on the optical properties is elucidated by means of structural, morphological, and optical analyses. SR‐GIXRD is used to study the NPs crystalline phase, while a combined Scanning Electron Microscopy (SEM) and Atomic Force Microscopy (AFM) analysis is employed to demonstrate the ability to control the size of the NPs and unveil the formation mechanism. Finally, High‐Angle Annular Dark Field STEM (HAADF‐STEM), XPS and transmission Fourirer Transfor Infrared (FTIR) spectroscopic investigations allowed us to track the evolution of the Sn oxide shell, its contribution to the stabilization of α‐Sn, and the tunability of the ultranarrow bandgap of α‐Sn as a function of the size Our findings suggest that quantum confinement is the primary mechanism enabling bandgap opening in α‐Sn NPs within the terahertz range.

## 2 Results and Discussion

### 2.1 Morphological and Structural Analysis: NPs Formation and α‐Sn Stabilization

The process used to stabilize α‐Sn on Si wafers consists of a Sn film deposition by physical evaporation, followed by thermal treatment (baking) up to 400 °C, free cooling in vacuum and finally a MW irradiation in Ar atmosphere. Details are reported in the Experimental Section.^[^ ^43^, ^44^ ^]^

To investigate the correlation between Sn film thickness and NP size, Sn films with nominal thicknesses of 4, 5, and 9 nm were deposited on Si substrates. Samples with 4, 5, and 9 nm ‐thick Sn films are labeled as Sn4, Sn5, and Sn9, respectively. The samples that underwent all the fabrication steps (baking/cooling and MW irradiation) are labeled as Snx_MW (with x = 4, 5, or 9), while the samples that stopped treatments before MW irradiation are labeled as Snx_bak.

**Figure 1:**
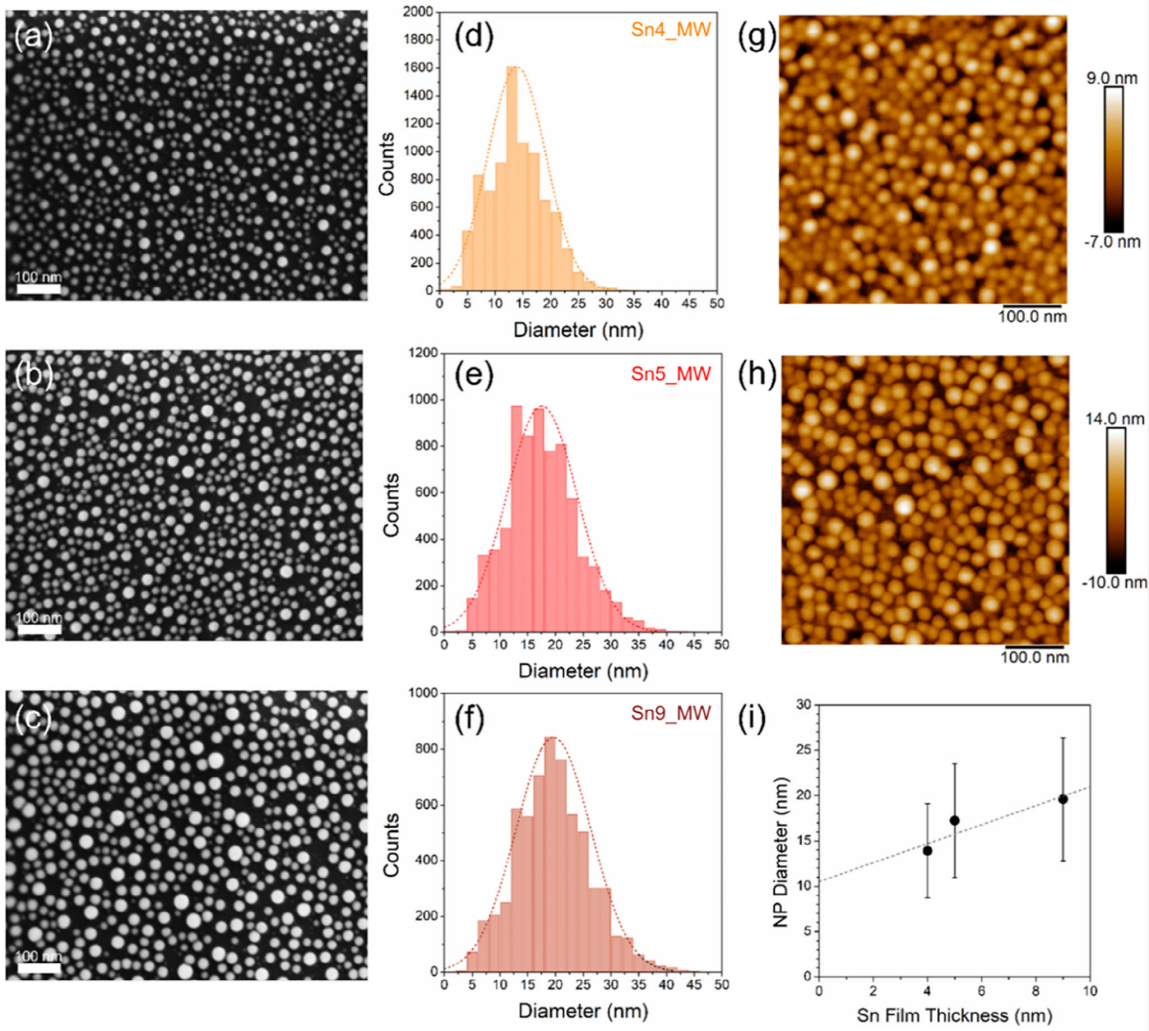
Morphological investigation of NPs obtained from Sn films of different thickness: Effect of microwave irradiation. a–c) SEM images of samples Sn4_MW, Sn5_MW, and Sn9_MW along with the corresponding size distributions d–f). AFM topography of g) Sn4_MW and h) Sn5_MW samples and i) NPs diameter vs deposited Sn film thickness.

**Figure 2:**
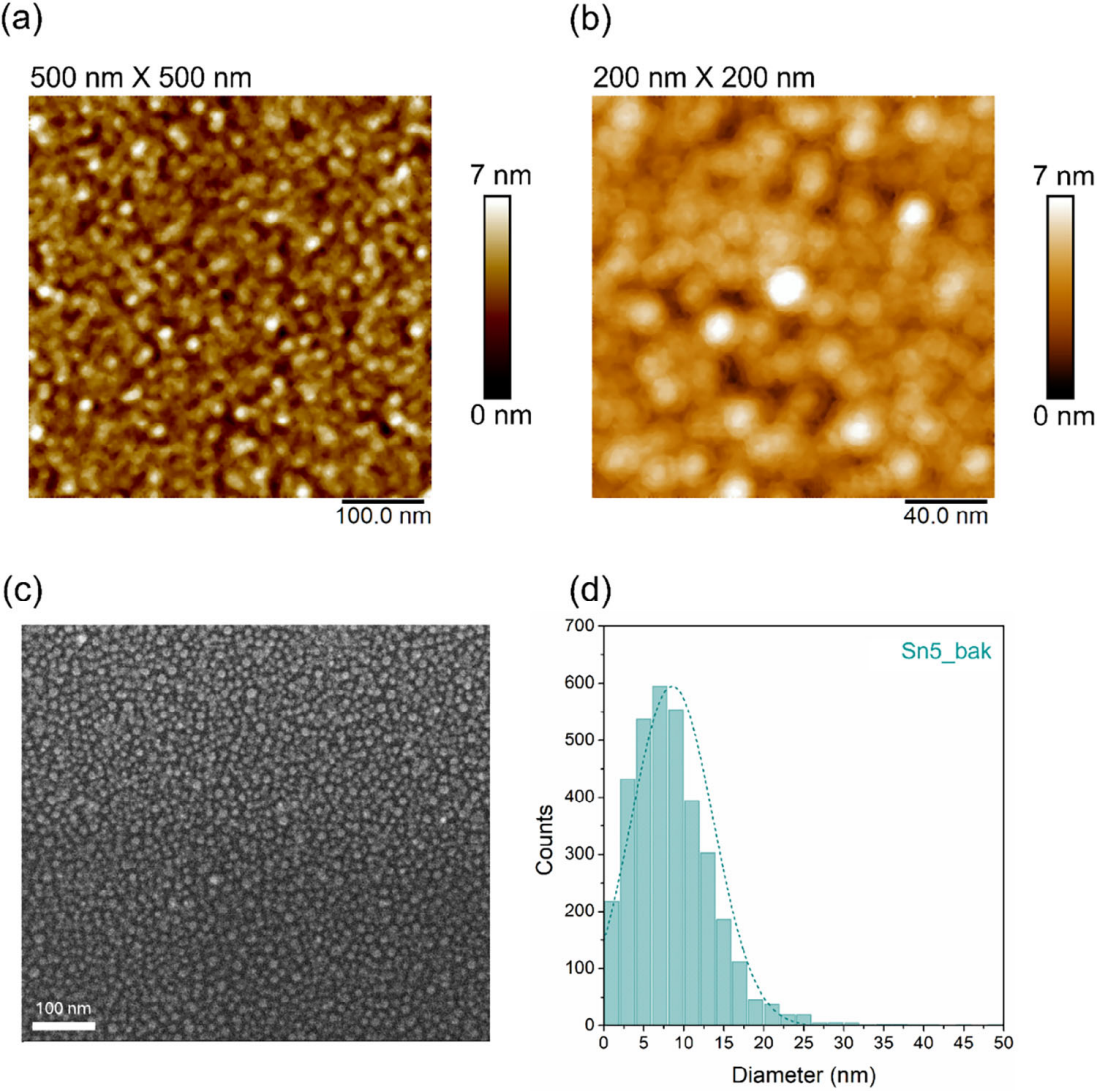
The baking process effect on the NPs formation. a,b) AFM, c) SEM, and d) size distribution of Sn5_bak sample after the baking process.

To estimate the NP size, SEM analysis was conducted on the MW‐irradiated samples (**Figure** **1**a–c). The NP size distributions were fitted by Gaussian curves yielding average diameters of 14, 17, and 20 nm for the samples Sn4_MW, Sn5_MW, and Sn9_MW, respectively (Figure 1d–f). The AFM analysis performed on the Sn4_MW and Sn5_MW samples (Figure 1g,h, respectively) confirms the average sizes of the NPs, also allowing an evaluation of the NP height with respect to the substrate (Figure S1, Supporting Information). As shown in Figure 1i, despite the dispersion reflected in the standard deviation of Gaussian fits, a nearly linear relationship can be inferred between the average NP diameter and the deposited Sn film thickness, which suggests the possibility to control the average size via film thickness adjustment. The dewetting of metal thin films has been modeled in previous literature considering two different processes: 1) the hole nucleation and growth, typically encountered in the case of polycrystalline metal films,^[^ ^45^, ^46^, ^47^ ^]^ and 2) the spinodal dewetting.^[^ ^48^, ^49^ ^]^ The former predicts a linear dependence between the NPs size (*d*) and the film thickness (*t*) while the latter is described by a 𝑑 ∝ 𝑡^5⁄3^ power‐law relationship. Based on the measured linear trend of *d* versus *t*, a hole nucleation and growth mechanism could be suggested, but the extrapolation of the fitting straight line inconsistently predicts NPs with a diameter of ≈10 nm in the limit of vanishing *t*. This can be explained considering that the process consists of a combination of thermal, cooling, and MW irradiation treatments that cannot be described by a single dewetting process. To evaluate the effect of the single steps, for one of the samples (Sn5_bak) the process was stopped after the baking, letting the temperature to reach freely the room value (in vacuum condition). The SEM and AFM analyses performed at the end of the baking/cooling step revealed the presence of tiny NPs of ≈8 nm in place of the uniform Sn film (**Figure** **2**). Based on the large difference of size with respect to the corresponding sample treated also with the MW, it is clear that the MW irradiation provides an appreciable size increase, likely due to a further temperature‐induced coalescence of nanoparticles. Indeed, it is well known that MW irradiation induces extremely rapid heating and cooling^[^ ^50^ ^]^ resulting into an almost instantaneous change of the surface temperature from ≈200–270 °C and vice versa.^[^ ^44^ ^]^ The presence of neck structures between the NPs detected by means of high‐resolution AFM investigations (Figure S2, Supporting Information) supports the coalescence between the tiny NPs formed during the baking step as the main process for the formation of larger NPs during the MW irradiation. It is worth mentioning that the process is independent of the kind of Si wafer employed, as the same behavior is observed on heavily doped, lightly doped, and intrinsic substrates.

**Figure 3:**
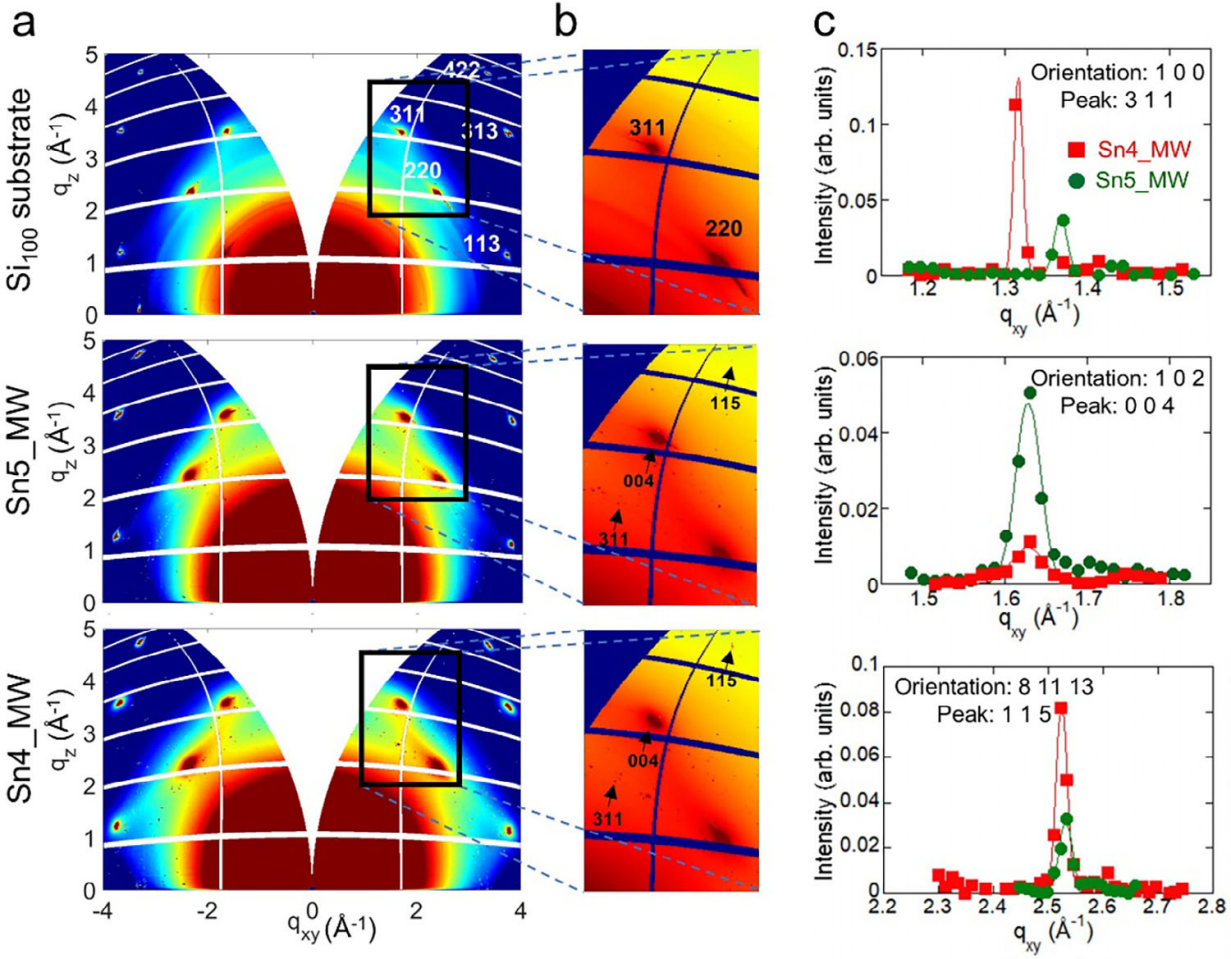
α‐Sn crystalline structure identification. a) GIXRD images of the Si(100) substrate (upper), Sn5_MW sample (middle) and Sn4_MW sample (lower). The Bragg reflections corresponding to Si are indexed in the upper panel. b) Magnified GIXRD images centred ≈220 of the Si(100) substrate. Resolution‐limited peaks associated with highly crystalline cubic α‐Sn grains can be identified in the magnified images of Sn5_WM and Sn4_WM samples. c) Background‐subtracted GIXRD profiles of Sn4_MW (red squares) and Sn5_MW (green circles), integrated along the 𝑞_𝑧_ direction, indicating (311), (004), and (115) reflections marked by black arrows in (b). The ℎ𝑘𝑙 indices and respective orientations, are indicated.

To investigate the crystalline structure of the NPs, SR‐GIXRD measurements have been carried out, orienting the substrate along the (100) crystallographic direction and using X‐ray synchrotron source at ELETTRA (12.4 keV). The SR‐GIXRD images shown in **Figure** **3** refer to the GIXRD images of the Sn4_MW and of the Sn5_MW samples, in addition to images of the bare Si (100) substrate. GIXRD measurements performed on samples Sn4_MW and Sn5_MW reveal the presence of weak diffraction features attributable to α‐Sn nanoparticles deposited on Si(100). These features appear as faint peaks surrounding the intense reflections of the silicon substrate and are not directly visible in the integrated diffraction intensity *I*(*q*), due to both their low intensity and the overwhelming background from Si scattering. To overcome this limitation, we focused on selected *q_xy_* cuts to isolate and analyze individual weak reflections. By means of GIDVis software package,^[^ ^51^ ^]^ three distinct α‐Sn (cubic *Fd‐3m*) reflections, (311), (004), and (115) were identified and analyzed in both samples. From the measured *q*‐values, we calculated the corresponding 2θ angles and cubic lattice parameters, assuming a monocrystalline structure and specific crystallographic orientations.

The extracted lattice parameters span the range 6.47–6.58 Å, consistent with the literature value for bulk α‐Sn (6.47 and 6.489 Å for ICSD#53789 and ICSD#40039, respectively) and suggest a small distribution of strain states or slight compositional variations among the nanoparticles. The corresponding 2θ values for each reflection, calculated using λ = 1.000 Å, are also reported together with the lattice parameters in Table S1 (Supporting Information). These results indicate that the α‐Sn NPs are structurally coherent and exhibit well‐defined crystallographic orientations, albeit with a small distribution of lattice parameters. This dispersion likely reflects local variations in particle size or interface conditions with the Si substrate. The presence of multiple reflections further supports the interpretation of a population of oriented monocrystals rather than a random polycrystalline phase.

No evidence of β‐Sn phase was observed. A few reflections corresponding to additional phases, such as SnO_2_, are also present.

### 2.2 Spectroscopic Investigation: α‐Sn Ultranarrow Bandgap Opening

**Figure 4:**
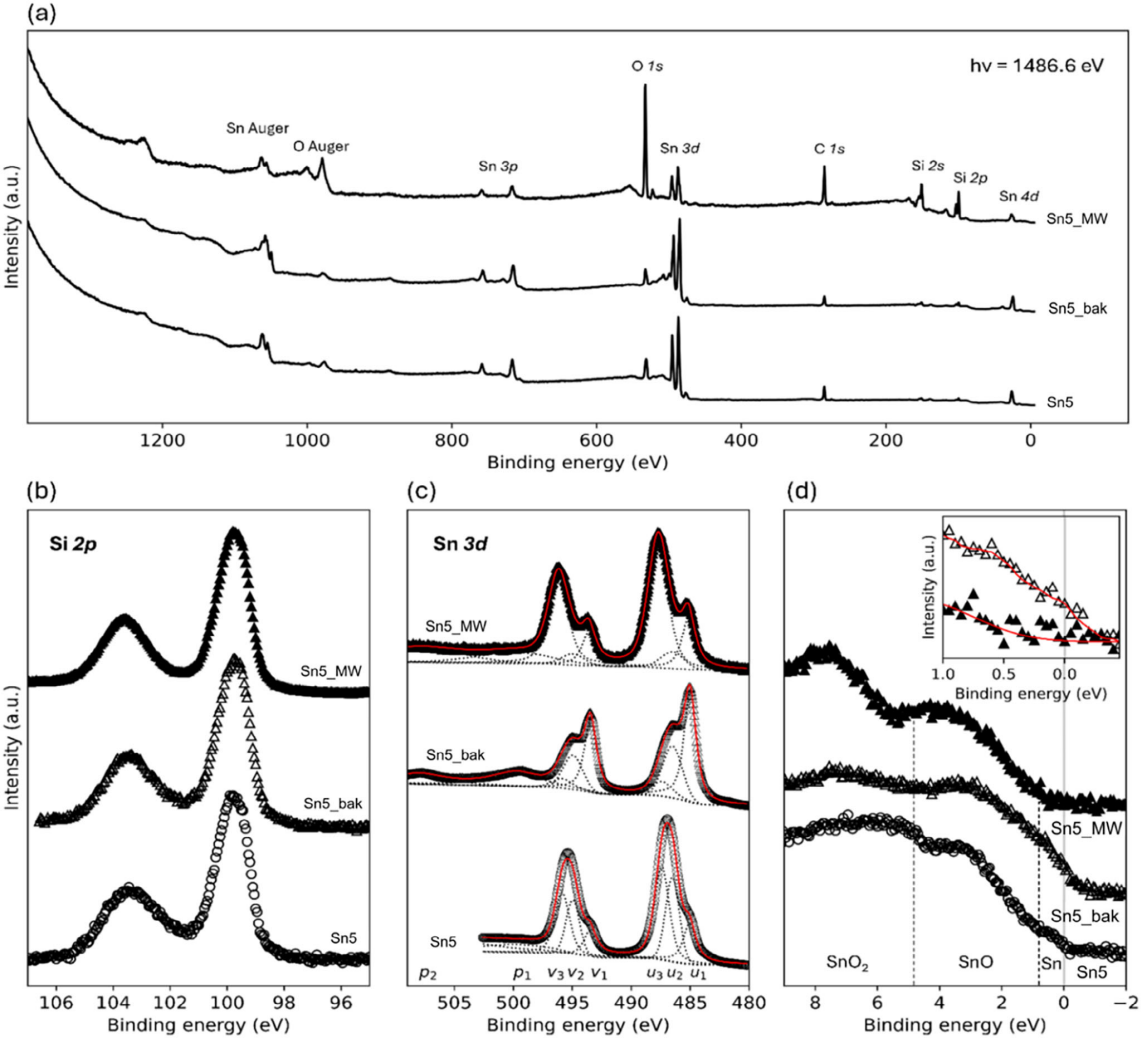
XPS analysis: Sn oxides evolution and non‐metallic nature of the Sn core. a) XPS survey scans of baked (Sn5_bak) and microwave‐irradiated (Sn5_MW) samples are shown together with the Sn5 reference sample. b) Si *2p* XPS; c) Sn *3d*; and d) valence band spectra are shown for the samples at the three stages. The Sn *3d* XPS spectra are deconvoluted in three components (dotted lines) and assigned to Sn (*u_1_,v_1_*), SnO (*u_2_,v_2_*), and SnO_2_ (*u_3_,v_3_*). The inset of panel d) is a zoom‐in of the valence states near the Fermi level of Sn5_bak (open symbols) and Sn5_MW (full symbols) samples to highlight the non‐metallic nature of the latter.

To elucidate the nature of the nanostructures and the impact of each fabrication step, XPS measurements were performed on Sn5 samples at three process stages: as‐deposited (Sn5), post‐baking (Sn5_bak), and post‐MW irradiation (Sn5_MW). First, survey spectra allowed to identify the core orbitals and the atomic species at the surface, as labeled in **Figure** **4**a. Considering the surface sensitivity of the photoemission process, the presence of Si signals from the substrate can be justified owing to morphological defects at the nanoscale, while the occurrence of O and C is usually associated with adventitious impurities and/or the formation of chemically bonded states during the synthesis or the exposure to the atmosphere.

Interestingly, the MW‐irradiated sample Sn5_MW exhibits a rather intense signal from Si, C, and O orbitals, suggesting that the MW treatment may favour further clustering and/or coalescence processes, leaving a larger portion of the substrate exposed to the probing x‐ray photons, in agreement with the SEM and AFM investigations (see Figure 2). To assess the possible role of the Si substrate in the stabilization of the α‐phase, XPS spectra of Si *2p* were measured (Figure 4b). The two broad spectral features ≈100 and 103 eV indicate the presence of Si^0^ and Si^3+^/Si^4+^ species, respectively, suggesting the coexistence of different Si oxides at the surface of the Si substrate. As expected, none of the relevant spectral feature changes across the sample series, since those signals refer to the substrate surface portions among particles. On the other hand, the analysis of the Sn core levels offered valuable insights into the effects of the different treatments. We performed a peak‐fitting analysis on Sn *3d* spectra of all samples modeling their spectral shape with three doublets, comprising the Sn *3d_3/2_* and Sn *3d_5/2_* contributions, separated by a spin‐orbit split of 8.45 eV and reported in Figure 4c as *u_i_* and *v_i,_* with *i* = 1,2,3. The three components (dotted lines in the figure) are assigned to Sn (*u_1_*,*v_1_*), SnO (*u_2_*,*v_2_*), and SnO_2_ (*u_3_*,*v_3_*). Specifically, doublet 1 accounts for metallic Sn^0^, while doublets 2–3 take into account Sn^2+^‐Sn^4+^ contributions, aiming to model the Sn oxides SnO and SnO_2_, which possibly constitute the NPs shell in the Sn5_bak and in the Sn5_MW samples. Two additional singlet contributions (*p_1_* and *p_2_*) have been included to model two plasmon peaks, allowing a proper background subtraction as well.

As one can see in the figure, Sn5 sample exhibits a similar amount of SnO and SnO_2_ (486.5 and 487.4 eV), together with a much smaller signal of metallic Sn (485.07 eV), possibly owing to the presence of a surface oxide. While these ratios agree with previous reports,^[^ ^52^ ^]^ the spectral shape of Sn5_bak sample appears rather different. First, the contribution of SnO_2_ is almost suppressed while the metallic Sn° contribution has a much larger intensity ratio, allowing to identify its binding energy at 484.99 eV. In order to rationalize this evolution of the spectral shape, we can consider for the NPs in sample Sn5_bak an oxidation gradient Sn‐SnO‐SnO_2_ from the metallic core to the outer shell layers, as reported in refs. [53, 54]. Experimental and theoretical evidence pointed out that the oxidation process during continuous heating mostly yields SnO in the considered temperature range^[^ ^55^, ^56^ ^]^ (even if the NP size can sensitively change the oxidation behavior^[^ ^57^ ^]^). Hence, in our baked samples we may expect the outer SnO_2_ shell to be disrupted by the heat treatment, leaving the NP with a shell mostly composed of SnO. As for the more intense Sn^0^ signal from the metallic core, we can consider the possibility that such process induces an average thinner oxide layer when the dewetting process leads to the NPs formation.

**Figure 5:**
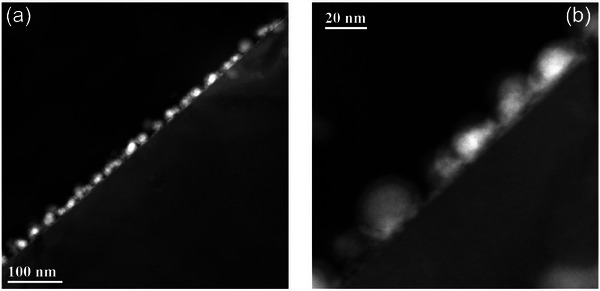
Cross‐sectional HAADF‐STEM images of Sn5_MW sample.

After MW irradiation, the spectral shape of Sn *3d* XPS spectra sustains a further considerable change. Surprisingly, in Sn5_MW sample, the larger spectral contribution comes from Sn^4+^ peak, while the intermediate SnO oxide ratio appears strongly reduced, suggesting that the MW irradiation favours the stabilization of SnO_2_ as NPs shell. However, we also observe a clear binding energy shift for both Sn^0^ (485.15 eV) and Sn^4+^ (487.75 eV) contributions. The higher binding energy of the Sn contribution can be interpreted as a signature of the α‐Sn phase stabilization in the NPs core, as previous XPS studies^[^ ^58^, ^59^ ^]^ indicate an energy shift of ≈0.3 eV with respect to β‐Sn phase. Here, the observed energy shift appears ≈0.2 eV and the above interpretation is justified considering lower energy resolution in the present data. On the other hand, we can interpret the peculiar behavior of Sn^4+^ as a result of the possible stabilization of a crystalline phase of SnO_2_, as the observed energy shift is compatible with the reported Sn *3d* XPS spectra of amorphous and crystalline SnO_2_.^[^ ^60^ ^]^ Earlier structural analyses on Sn nanostructures report an amorphous SnO‐SnO_2_ shell for as‐deposited samples,^[^ ^53^, ^61^ ^]^ while the heating process mostly stabilizes the crystalline phase of SnO oxide in its early stages (200–400 °C), a mixture of SnO‐SnO_2_ in the range 500–700 °C, and finally only crystalline SnO_2_ is detected above 800 °C.^[^ ^55^, ^62^ ^]^ Even if the oxidation process can be strongly dependent on the NPs size,^[^ ^57^ ^]^ it is apparent that the crystalline phase of SnO_2_ mostly occurs at high temperatures. In this context, the MW irradiation may act as an intense annealing process locally delivering an energy corresponding to an annealing treatment at a temperature much higher than that measured by the pyrometer in the MW‐chamber (≈270 °C). The crystallization process of SnO_2_ shell has already been documented for both high temperature annealing and microwave treatment, which can be a further cause for the observed increase of NPs size after MW irradiation.^[^ ^62^, ^63^ ^]^ In order to confirm the core‐shell structure suggested by the XPS analysis and by previous works on Sn NPs, representative HAADF‐STEM images were collected on the Sn5_MW sample (**Figure** **5**) prepared in cross‐section. The images confirmed the formation of NPs composed of a core‐shell structure composed of Sn core and an oxide shell, with a shell thickness of ≈5 nm.

From the XPS analysis, it is also interesting to note that the metallic transition of the Sn core from the β‐ to the α‐phase is accompanied by the complete transition from a major SnO character to a major SnO_2_ character of the shell. The final stabilization of α‐Sn core and the crystalline SnO_2_ shell after the MW treatment confirms suggestions of the SR‐GIXRD analysis.

Based on these findings, a possible mechanism for the stabilization of the α‐Sn can be proposed. It is well known that the oxidation of metal NPs can induce a large tensile strain on the metallic core,^[^ ^64^, ^65^, ^66^ ^]^ leading in some cases to the formation of holes.^[^ ^64^ ^]^ Moreover, during the cooling after the MW heating step the core could experience a volume contraction more pronounced than the outer shell, due to the different expansion coefficients of the metallic core and the oxide shell. This could result in a tensile strain in the metallic β‐phase core. Finally, the strain energy could be relaxed via a phase transition from β‐Sn to α‐Sn,^[^ ^67^ ^]^ favored by the larger volume of the α‐Sn phase compared to the β‐Sn phase (≈27% volume increase^[^ ^19^ ^]^).

Figure 4d shows the XPS valence spectra of all samples. According to previous photoemission studies,^[^ ^53^, ^68^ ^]^ we labeled three energy regions on the basis of the Sn phase which contributes most to the spectral weight. The electronic states near the Fermi level (up to a few hundred meV) belong to metallic Sn, while the density of states is mostly attributed to Sn *5s* and Sn *5p* hybridized orbitals of SnO in the range from ≈1–4 eV. Finally, at energy beyond 5 eV O *2p* states of SnO_2_ play the most important role. The behaviour of O *2p* offers a further heuristic argument to support the SnO_2_ crystallization scenario, in agreement with the SR‐GIXRD analysis, since it appears as a very broad feature ≈6 eV in the Sn5 sample, while in Sn5_MW sample its profile tends to sharpen and to shift ≈7.5 eV. On the other hand, in Sn5_bak the position of this feature is apparently aligned with the one of Sn5_MW, but it is strongly suppressed, suggesting an early coexistence of crystallizing SnO_2_ and SnO. In Sn5_MW sample, the opening of a small gap is observed, while the heat treatment appears to enhance the density of states at the Fermi level in Sn5_bak sample, which is possibly at the origin of the strong plasmon signal in the corresponding Sn *3d* spectrum. In the inset of Figure 4d, we can closely observe the suppression of the states near the Fermi level in the Sn5_MW sample (full symbols) with respect to the Sn5_bak (open symbols), confirming the occurrence of a gap opening. As the range of suppressed states only involves metallic Sn, we may address the gap opening as a further effect of the strain on the metallic NPs core or to a quantum confinement effect. In normal conditions, α‐Sn exhibits a semimetal electronic structure, therefore it is possible that the very few electronic states near the Fermi level are highly susceptible to external perturbations. Previous studies on strained α‐Sn grown on InSb have demonstrated that the application of strain can induce a topological gap of ≈50 meV^[^ ^69^ ^]^ and its non‐trivial topological properties have been extensively explored under both compressive and tensile strain conditions.^[^ ^31^, ^34^ ^]^ On the other hand, it is well known that nanostructures can also experience a strong change in the optical behaviour as a function of the strength of quantum confinement. Nevertheless, to the best of our knowledge, there is no experimental report about the effect of size on the optical properties of α‐Sn nanocrystals.

The optical properties of the samples were investigated using FT‐IR spectroscopy collecting transmission signals in the Mid‐InfraRed (MIR) and Far‐InfraRed (FIR) ranges and calculating the optical density (*OD*) as *OD* = − *log*_10_(*I_sample_*/*I_bare_*), where *I_sample_* is the intensity of the radiation transmitted by the sample and *I_bare_* is the intensity of the radiation transmitted by the bare silicon substrate. It is important to note that, since the absorption intensity is influenced by the fraction of the sample surface covered by nanoparticles (filling factor), the optical density measurements are subject to a systematic error caused by sample non‐uniformity.^[^ ^70^ ^]^ However, SEM analysis revealed that the samples used in the FTIR measurements have nearly identical filling factors of ≈0.4, which we verified to have a negligible impact on the estimated energy gap values.

**Figure 6:**
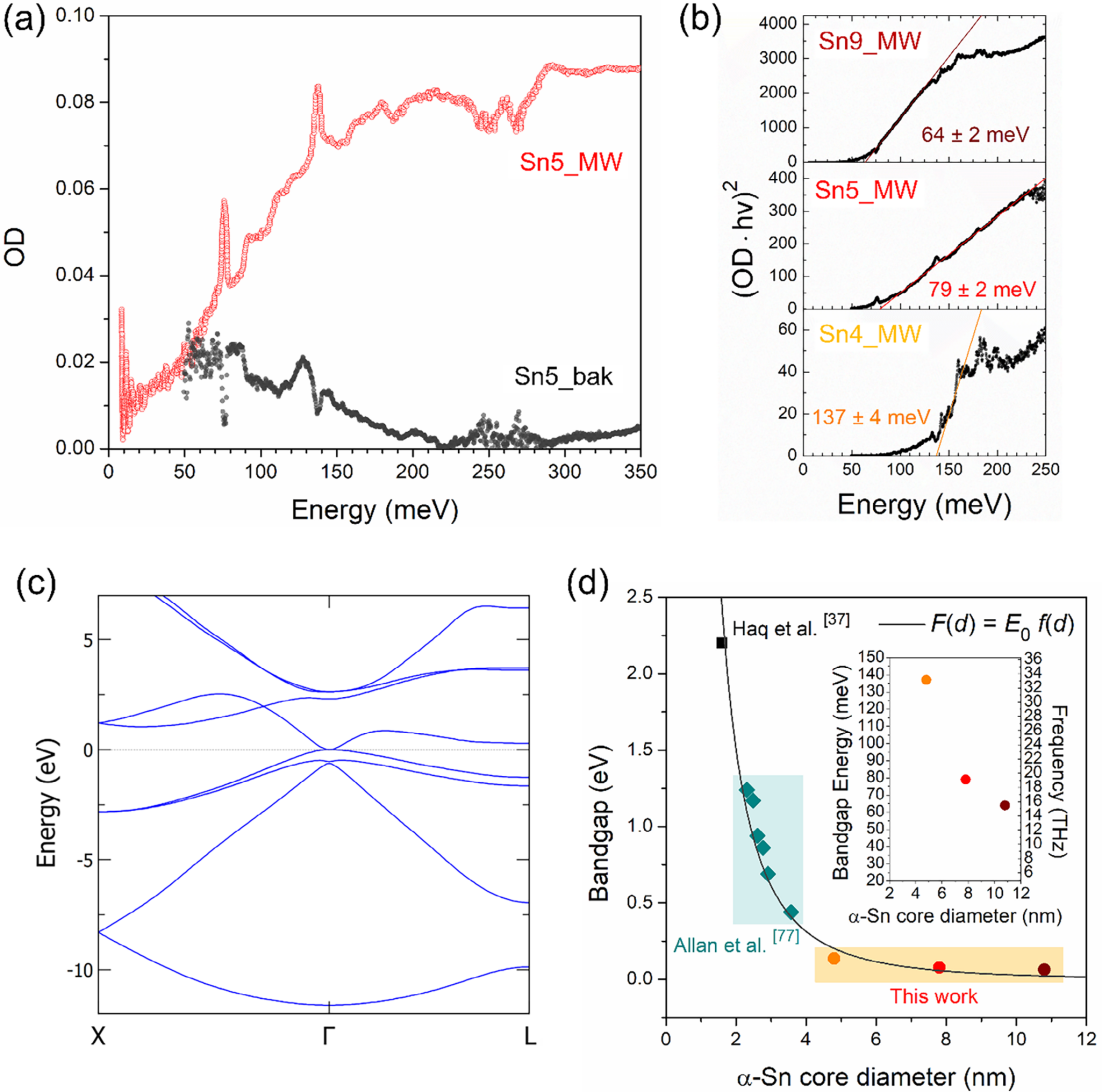
Bandgap tuning in the THz range: Quantum confinement in α‐Sn NPs. a) Optical density of the absorption spectra of Sn5_bak and Sn5_MW samples. b) Tauc plot of the samples and corresponding bandgap values. c) Experimental values of bandgap of α‐Sn particles extracted for average diameters of 4.8, 7.8, 10.8 nm (this work) and 1.8 nm (from Ul Haq et al.^[^ ^37^ ^]^) along with the theoretical values calculated by Allan et al.^[^ ^77^ ^]^ The fitting curve is derived from the QC model proposed in ref. [78]. d) DFT band structure of bulk α‐Sn from which electron and hole effective masses have been extracted. The dashed line indicates the Fermi level position.

The *OD* spectrum of all MW‐irradiated samples displays a sigmoidal profile, indicating the onset of inter‐band electronic transitions in the FIR. Conversely, the spectrum of the non‐irradiated samples after the baking step do not show any significant increase in the optical density signal in the explored energy range (see, as an example, the comparison between the *OD* profiles of the Sn5_bak and Sn5_MW samples in **Figure** **6**a). This result is consistent with both the SR‐GIXRD and XPS analysis performed on the same samples, confirming the key role played by the MW irradiation for the stabilization of the semiconductive α‐Sn phase.

The well‐known Tauc‐plot method^[^ ^71^, ^72^, ^73^, ^74^, ^75^ ^]^ was employed to estimate the energy of the bandgap *E_g_* of the semiconducting samples, using the relation (*OD*  ·  *h*ν)^2^  =  *B*(*h*ν  −  *E_g_*). The *E_g_* value for each sample was then obtained by fitting the linear part of the curve in an appropriate range around the inflexion point and extrapolating the energy at (*OD*  ·  *h*ν)^2^  =  0, as shown in Figure 6b. Bandgap values of 64, 79, and 137 meV were estimated for the Sn9_MW, Sn5_MW, and Sn4_MW samples, respectively, corresponding to a frequency window from 15 to 35 THz.

### 2.3 Size‐Dependent Bandgap Tuning of α‐Sn NPs: A Quantum Confinement Perspective

FTIR spectroscopy clearly indicates a bandgap widening as the NP size decreases. To assess a possible role of quantum confinement (QC) effects, it is necessary to compare the size of the NP core with the Bohr exciton radius $a_{B}^{*}=\varepsilon_{\infty }a_{BH}^{*}/\mu$ in α‐Sn, where ɛ_∞_ is the high‐frequency relative permittivity of the material, $a_{BH}^{*}=0.529$Å is the Bohr radius of the hydrogen atom and $\mu =\frac{m_{e}^{*}\;m_{h}^{*}}{m_{0}\left(m_{e}^{*}+m_{h}^{*}\right)}$ is the reduced mass normalized to the free electron mass *m*_0_. Due to the presence of the low‐permittivity SnO_2_ shell, a reduction of the screening effects is expected in the nanoparticle core.^[^ ^76^ ^]^ For analogous systems, it has been reported that the oxide shell thickness around the metallic Sn core is constant and approximately equal to 4.6 nm.^[^ ^61^ ^]^ The HAADF‐STEM images shown in Figure 5 agree well with this oxide shell thickness value, therefore, we evaluate the α‐Sn core radius by subtracting 4.6 nm from the total radius of the NPs. Under these hypotheses, we estimate in our samples an α‐Sn core radius in the range from 2.4 to 5.4 nm and ɛ_∞*eff*_ =  14, intermediate between that of α‐Sn (ɛ_∞_ =  24) and of SnO_2_ (ɛ_∞_ =  2.07). The electron effective mass in the lowest conduction band ($m_{e}^{*}$) and the hole effective mass in the highest valence band ($m_{h}^{*}$) were estimated from the DFT band structure (see Figure 6c). The electron effective mass was found to be isotropic and equal to ≈ 0.04 𝑚_0_, while the hole effective mass is markedly anisotropic, with an average value of ≈ 0.2 𝑚_0_, obtained as the harmonic mean of the effective mass tensor principal values. Using the previous values, a Bohr exciton radius value of ≈22 nm is obtained. This value is to be compared with the radius of the α‐Sn core of the NPs. As mentioned, in our samples a core radius in the range between 2.4 and 5.4 nm was estimated, much smaller than the Bohr exciton radius. These results suggest that QC effects could be strong and play a major role in determining the bandgap value. To further investigate this aspect, we report in Figure 6d our bandgap estimates versus the size of the NP core, together with the experimental data by Haq et al.^[^ ^37^ ^]^ and the theoretical calculations by Allan et al.,^[^ ^77^ ^]^ based on quantum confinement effects on smaller quantum dots (QD). These bandgap energies were fitted with a semiempirical sizing function of the type *F* (*d*) =  *E*_0_ *f*(*d*),^[^ ^78^ ^]^ where *d* is the NP core diameter, *E*_0_ is a fitting constant, approximately corresponding to the value of the bandgap for $d=a_{B}^{*}$ and

(1) $$f\;\left(d\right)=e^{-d/a_{B}^{*}}/{\left(1-e^{-d/a_{B}^{*}}\right)}^{2}$$

This is a simplified version of the formula proposed in ref. [78] since the dependence of the non‐parabolicity effects on the QD size is neglected. However, it maintains the correct limits for weak ($d\gg a_{B}^{*}$, f→0) and for strong ($d\ll a_{B}^{*}$, $f\approx {\left(a_{B}^{*}/d\right)}^{2}$) confinement regimes. Figure 6d shows that the fitting function can reasonably approximate all the considered data in the diameter range from 1.6 to 11 nm, further supporting the interpretation of the bandgap evolution as a function of the NP size as a QC effect.

## 3 Conclusion

In an upside‐down view with respect to the closing of the Si and Ge bandgap conventionally employed to achieve redshifted absorption in the mid‐IR range, in this work, we demonstrated the possibility to open and control an ultranarrow bandgap in the 15–34 THz range by tuning the α‐Sn NPs size. Sn NPs with α‐phase cores were synthesized and stabilized at room temperature on silicon substrates using a CMOS‐compatible process based on baking of uniform tin films and subsequent irradiation of the NPs by microwaves. The ability to control the average size of NP distribution was also demonstrated. Synchrotron radiation GIXRD revealed the presence of the α‐phase only in the samples irradiated by MW, while XPS analysis suggested that the transition of the Sn core from the metallic β‐ to the semiconducting α‐phase is accompanied by the formation of a SnO_2_ shell. The latter may also be instrumental in triggering the phase transition from β to α‐Sn. FTIR spectroscopy revealed direct interband transitions in the far infrared spectrum, with optical bandgaps ranging between 64 and 137 meV for NPs with average diameter of the α‐Sn core from ≈5 to ≈11 nm. The relationship between the optical bandgap and the α‐Sn particle size is consistent with a quantum confinement effect suggesting the possibility of significantly expanding the covered frequency range by better controlling the NPs size distribution. The full compatibility of the fabrication process with CMOS technology provides the opportunity to develop integrated solutions and can pave the way toward significant advancements in THz technology.

## 4 Experimental Section

### Procedure for the α‐Sn NPs Formation on Si

The process employed to stabilize α‐Sn on Si wafers consists of four steps:^[^ ^43^, ^44^ ^]^ i) deposition of a Sn film by physical evaporation, ii) thermal treatment (baking) up to 400 °C with a temperature ramp of 3800 °C per hour, followed by a iii) free cooling in vacuum down to 200 °C and finally iv) a 450 W 3 min‐long MW irradiation in Ar atmosphere. Si substrates used in this work were lightly p‐doped (however, the process was independent of the Si doping). No HF dip treatment was performed before depositing the Sn film.

### Morphological Investigation

The morphological investigation was performed by means of SEM and STEM. SEM (AURIGA and ∑igma, Zeiss) analysis was performed working between 3 and 8 kV, using InLens detector to collect secondary electrons in high vacuum condition. The distributions were analyzed more than 6000 NPs for each sample. Jeol JEM‐2200FS TEM equipped with Schottky (thermal field emission) emitter, HAADF–STEM detector and in‐column Omega filter was used. The accelerating voltage was set at 200 kV, and the samples were mounted on double‐tilt holders; images were obtained in HAADF‐ STEM mode. Samples were prepared to be observed in cross‐section. They were sectioned by diamond saw and then mechanically grinded and polished down to 20 micron thickness. The nanoparticles/silicon chip interface was finished with Ar‐ion milling (Gatan Model 691 PIPS) down to electron beam transparency.

AFM topography images were acquired in tapping mode using a Dimension Icon atomic force microscope (Bruker, Germany) in ambient conditions. A sharp AFM tip with 1 nm end radius fabricated on a cantilever with spring constant and resonant frequency of 0.12 N nm^−1^ and 100 kHz, respectively (Peakforce‐HIRS‐SSB, Bruker, Germany) was used. Images of the sample surface were collected at scan sizes of 900, 500, and 200 nm^2^ and scan rate 0.5 Hz.

### Structural Measurements: Synchrotron Radiation GIXRD

Grazing Incidence X‐ray Diffraction (GIXRD) measurements were performed using X‐ray synchrotron source at ELETTRA, Trieste at the crystallographic XRD1 beamline at the ELETTRA synchrotron facility in Trieste. The beam energy was set to 12.4 keV using a vertical collimating mirror and a double‐crystal Si(111) monochromator. Initially, the samples were aligned along the vertical *z*‐axis, and the grazing incidence angle was determined experimentally. The measurements were performed by rotating the sample around the *z*‐axis, which was perpendicular to the film surface, covering an angle of 180° with an exposure time of 20 s. A beam size of 0.2 × 0.2 mm^2^ was used. Diffraction images were collected using a Dectris Pilatus 2 M detector, which had 1475 × 1679 pixels with an area of 172 × 172 µm^2^. The sample‐detector distance was set to 150 mm. Diffraction data were calibrated using a LaB_6_ standard and analyzed with the GIDVis software package.^[^ ^51^ ^]^

### Spectroscopic Investigation: XPS and FT‐IR

All the XPS measurements were carried out at room temperature, exploiting an in‐house ultra‐high vacuum setup equipped with a double Al‐Mg anode SPECS XR50 X‐ray source and a multichannel Omicron EA125 electron analyzer, operating at base pressure <2 × 10^−9^ mbar. The Al Kα excitation line (hν = 1486.6 eV) was employed to probe the electronic structure of a sample series. All samples were maintained within the load‐lock chamber under a stringent vacuum regime (base pressure of 10^−7 ^mbar) for extended periods and a couple of hours in preparation chamber (base pressure of 10^−10 ^mbar) before the measurements.

The optical properties of the samples were investigated using FTIR spectroscopy (Bruker IFS 66 v/s) with a custom‐built setup in transmission configuration, placing the sample directly in front of the detector to minimize scattering effects due to the NP size and surface roughness of the substrate. Measurements were conducted in the Mid‐InfraRed (MIR) and Far‐InfraRed (FIR) ranges using a DTGS detector coupled with a KBr and a Multilayer Mylar beamsplitter, respectively.

### Computational Methods

DFT simulations were performed in a plane‐wave basis using the Quantum Espresso package.^[^ ^79^ ^]^ The Heyd–Scuseria–Ernzerhof exchange‐correlation functional^[^ ^80^ ^]^ and fully‐relativistic norm‐conserving Vanderbilt pseudopotentials without non‐linear core corrections were adopted. The plane‐wave energy cutoff was set to 50 Ry and the first Brillouin zone was sampled using a 12 × 12 × 12 Monkhorst‐Pack grid. The α‐Sn primitive cell was relaxed without constraints until interatomic forces were lower than 10–5 Ry/Bohr and the band structure was subsequently computed via interpolation on a maximally localized Wannier function basis.^[^ ^81^ ^]^ The effective mass tensors were obtained from the curvature of the highest valence band and of the lowest conduction band in Γ, evaluated within a second‐order finite‐difference approximation.

### Statistical Analysis

Nanoparticle sizes were measured from SEM images using ImageJ software. The images were converted to 8‐bit format, the Watershed algorithm was applied to separate touching nanospheres and the Analyze Particles tool was used to quantify the particles, with the “Exclude on Edges” option enabled to omit incomplete particles located at the image borders. NanoScope Analysis 3.0 software (Bruker) was used to process the AFM images. The only image correction applied was flattening. The particle size distribution was determined using the particle analysis tool within NanoScope Analysis 3.0 software. For both SEM and AFM analysis, the measured data were then exported to Origin (OriginLab) for statistical analysis. Histograms were plotted to visualize the particle size distribution, and descriptive statistics were calculated to obtain the mean particle size and standard deviation (SD). To further characterize the distribution, histograms were fitted with a Gaussian function, where the fitted mean and SD correspond to the average particle size and the width of the size distribution, respectively.

## Conflict of Interest

The authors declare no conflict of interest.

## Supporting information



Supporting Information

## Data Availability

The data that support the findings of this study are available from the corresponding author upon reasonable request.
