# Supplementary material for: Tuning Ultra‐Narrow Direct Bandgap in α‐Sn Nanocrystals: A CMOS‐Compatible Approach for THz Applications
Source: Small. 2025 Nov 2;21(50):e09166. doi: 10.1002/smll.202509166 (PMC12710133; doi:10.1002/smll.202509166)
Supplement: Supplementary file 1 — Supporting Information [file SMLL-21-e09166-s001.docx]

Supporting Information

**Tuning Ultra-Narrow Direct Bandgap in α-Sn Nanocrystals: A CMOS-Compatible Approach for THz Applications**

*Tiziano Bertoli, Elena Stellino, Francesco Minati, Camilla Belloni, Giovanni Tomassucci, Emanuele Bosco, Silvano Battisti, Leonardo Puppulin, Davide Cristofori, Vittorio Morandi, Francesca Rossi, Demetrio Logoteta, Alessandro Nucara, Luisa Barba, Gaetano Campi, Naurang Lal Saini, Fabrizio Palma, Pietro Riello, Michele Back*, Fernanda Irrera**


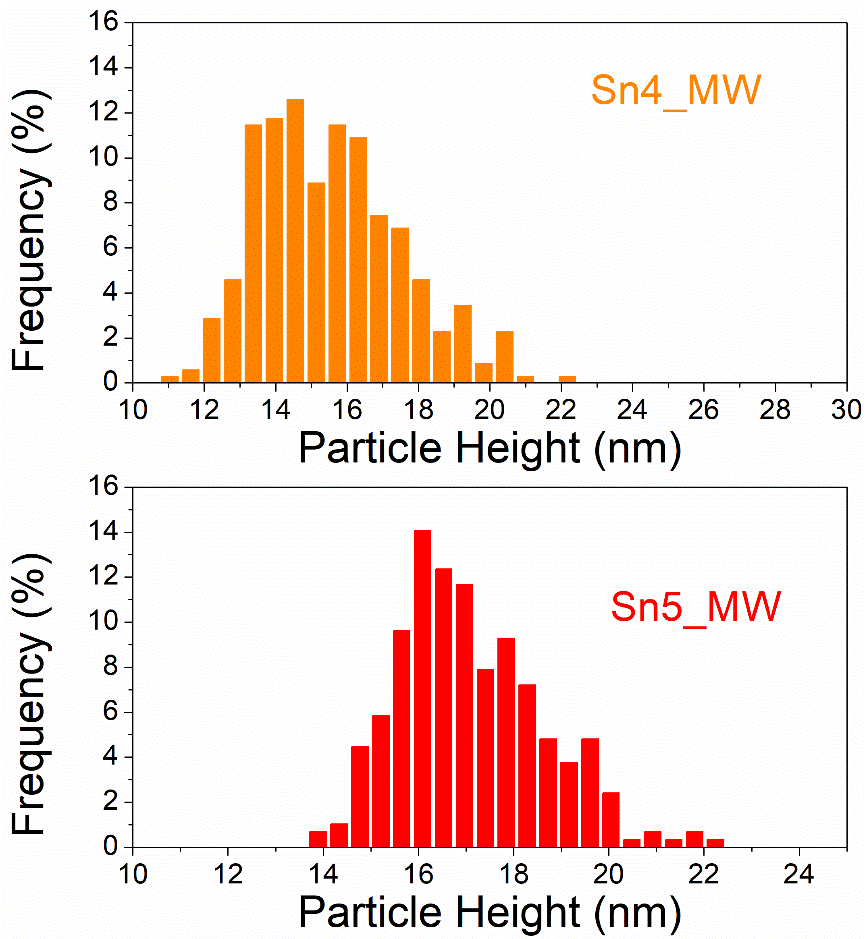


**Figure S1.** Size distribution along z-axis for the Sn4_MW and Sn5_MW samples estimated by means of AFM.


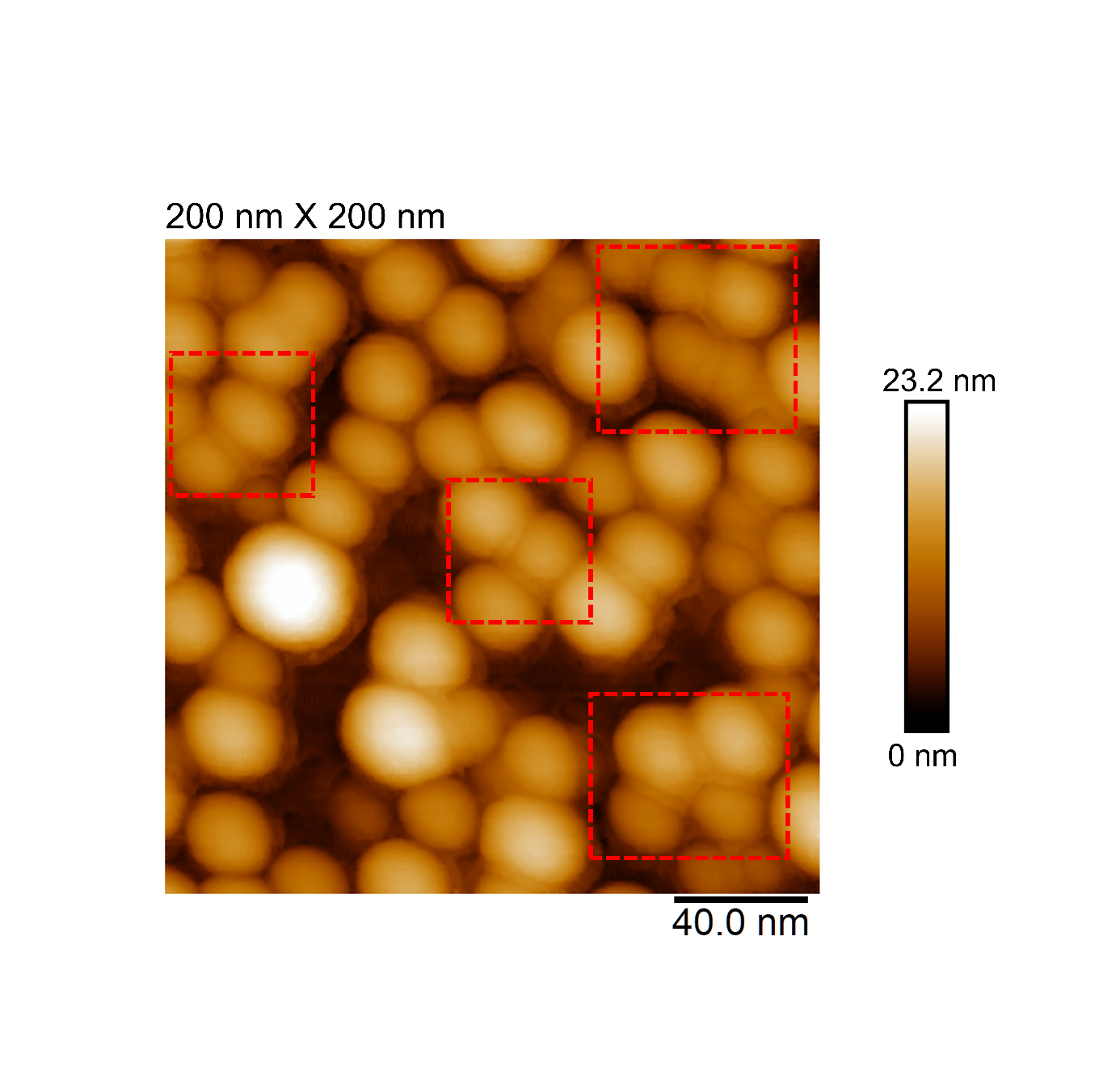


**Figure S2.** High resolution AFM image of Sn5_MW.

| **(hkl)** | **Sn5_MW**  **a (Å)** | **Sn5_MW**  **2θ (°)** | **Sn4_MW**  **a (Å)** | **Sn4_MW**  **2θ (°)** |
| --- | --- | --- | --- | --- |
| (311) | 6.58 | 29.19 | 6.49 | 29.64 |
| (004) | 6.47 | 35.90 | 6.47 | 35.90 |
| (115) | 6.51 | 47.05 | 6.50 | 47.18 |

**Table S1**. 2θ and lattice parameter extracted from GIXRD analysis of α-Sn nanoparticles on Si(100) (λ = 1.000 Å) in Sn4_MW and Sn5_MW samples. 2θ values are derived from $q$ using the relation $2\theta=2arcsin\left( \frac{\lambda q}{4\pi} \right)$, with λ = 1 Å.
